# Legal Notes for Institutional Workers

**Published:** 1911-10-28

**Authors:** 


### LEGAL NOTES FOR INSTITUTIONAL WORKERS.

**(The Editor will be pleased to answer legal queries in this column)**

### AN ATTACK UPON MEDICAL REFEREES.

Of the learned professions, the medical is that whose members seem to suffer most from the attacks of those who represent organised labour. The individual doctor is left alone—he could bring an action for libel. No specific instances of misconduct are cited: dates, names and other particulars would be difficult to prove. But the vaguest and vilest insinuations are often made, and, we regret to say, are frequently applauded. The latest exponent of this type of controversy is one Walter Barber, secretary of an organisation of twenty thousand trade-unionists. Speaking at a meeting in Bradford, this gentleman recently took occasion to attack all the certifying surgeons and medical referees in the Bradford district. He pointed out that the Act of 1907 provides that a man suffering from industrial disease must go before a medical referee, as the firm could dispute the certifying surgeon's decision. If correctly reported, he did not point out—as the fact is—that the workman, too, can appeal. This is one of those omissions which always characterise the utterances of your genuine agitator. He went on: "I say quite seriously and in the hope there may be pressmen here to take it down, that I am quite satisfied in my own mind that a large number of the medical fraternity in Bradford have sold themselves body and soul to the insurance companies, and it is with the utmost difficulty that we can get compensation for a workman suffering from an industrial disease. ('Shame!')" He then said that the trade unionists wanted the Act so altered that they could appeal against the decisions of medical referees.

### STATISTICS AS TO INDUSTRIAL DISEASES.

We ought to be content to treat this outburst with silent contempt; but, fortunately, the answer is ready to hand. It is contained in the statistics of Workmen's Compensation for 1910. They do not relate particularly with Bradford; but they speak volumes for the work of certifying surgeons all over the country. In 1910, 3,062 certificates of disablement were issued, while there were only 163 certificates of refusal to certify disablement. Large as is the number of certificates of disablement, the cases in which compensation was actually paid for industrial disease was much larger; while those who compile the statistics state that in some cases the employer does not require a formal certificate of suspension from the certifying surgeon before admitting the workman's claim. In some cases the certificate of the "works" doctor is accepted by the employer as sufficient. We venture to think that if the individual workmen who suffer from industrial disease in Bradford were canvassed, there would be ample evidence of careful and considerate treatment by the doctors who look after them.

### HINTS FOR THE LOCUM TENENS.

The man who has held junior hospital appointments often spends some time "doing locums" before he finds a practice of his own. The relations between practitioner and locum are sometimes clearly defined in a written agreement; but they are more often regulated by the custom of the profession. In the first place, if the engagement of a locum is made in London, the engagement commences from the time he leaves London, and the expenses are also reckoned from London. A locum tenens impliedly agrees to hand over all fees earned by him to his principal. He must not even retain a present given by a patient, nor a fee paid to him for giving evidence before a coroner or in a court of justice. He may, however, retain the out-of-pocket expenses incurred by attendance at court. With regard to travelling expenses, it is generally understood that the locum cannot be expected to walk more than a reasonable distance to see patients. The cost of the vehicles which he uses must be defrayed by the principal. A question which may vex the mind of a locum tenens is: Can I set up in practice in this neighbourhood when my time is up? In law there is no objection to his doing so, unless expressly forbidden by a written contract; but the code of medical ethics would strongly condemn such conduct. The mere fact, however, that a man was once a locum in a district would not prevent his setting up there for himself at some future time.

